# Adverse transverse-tubule remodeling in a rat model of heart failure is attenuated with low-dose triiodothyronine treatment

**DOI:** 10.1186/s10020-019-0120-3

**Published:** 2019-12-06

**Authors:** Shimin An, Nimra Gilani, Yuan Huang, Adam Muncan, Youhua Zhang, Yi-Da Tang, A. Martin Gerdes, Kaie Ojamaa

**Affiliations:** 1 Department of Biomedical Sciences, New York Institute of Technology College of Osteopathic Medicine, PO Box 8000, Northern Blvd., Old Westbury, New York, 11568 USA; 2 Department of Cardiology, State Key Laboratory of Cardiovascular Disease, Fuwai Hospital, Chinese Academy of Medical Sciences and Peking Union Medical College, 167 Beilishi Road, Beijing, 100037 China

**Keywords:** Heart failure, T-tubules, Triiodothyronine, Calcium transients, Contractility

## Abstract

**Abstract:**

Pre-clinical animal studies have shown that triiodothyronine (T3) replacement therapy improves cardiac contractile function after myocardial infarction (MI). We hypothesized that T3 treatment could prevent adverse post-infarction cardiomyocyte remodeling by maintaining transverse-tubule (TT) structures, thus improving calcium dynamics and contractility.

**Methods:**

Myocardial infarction (MI) or sham surgeries were performed on female Sprague-Dawley rats (aged 12 wks), followed by treatment with T3 (5μg/kg/d) or vehicle in drinking water for 16 wks (*n* = 10–11/group). After in vivo echocardiographic and hemodynamic analyses, left ventricular myocytes were isolated by collagenase digestion and simultaneous calcium and contractile transients in single cardiomyocytes were recorded using IonOptix imaging. Live cardiomyocytes were stained with AlexaFluor-488 conjugated wheat germ agglutinin (WGA-488) or di-8-ANEPPS, and multiple z-stack images per cell were captured by confocal microscopy for analysis of TT organization. RTqPCR and immunoblot approaches determined expression of TT proteins.

**Results:**

Echocardiography and in vivo hemodynamic measurements showed significant improvements in systolic and diastolic function in T3- vs vehicle-treated MI rats. Isolated cardiomyocyte analysis showed significant dysfunction in measurements of myocyte relengthening in MI hearts, and improvements with T3 treatment: max relengthening velocity (Vmax, um/s), 2.984 ± 1.410 vs 1.593 ± 0.325, *p* < 0.05 and time to Vmax (sec), 0.233 ± 0.037 vs 0.314 ± 0.019, *p* < 0.001; MI + T3 vs MI + Veh, respectively. Time to peak contraction was shortened by T3 treatment (0.161 ± 0.021 vs 0.197 ± 0.011 s., *p* < 0.01; MI + T3 vs MI + Veh, respectively). Analysis of TT periodicity of WGA- or ANEPPS-stained cardiomyocytes indicated significant TT disorganization in MI myocytes and improvement with T3 treatment (transverse-oriented tubules (TE%): 9.07 ± 0.39 sham, 6.94 ± 0.67 MI + Veh and 8.99 ± 0.38 MI + T3; sham vs MI + Veh, *p* < 0.001; MI + Veh vs MI + T3, *p* < 0.01). Quantitative RT-PCR showed that reduced expression of BIN1 (Bridging integrator-1), Jph2 (junctophilin-2), RyR2 (ryanodine receptor) and Ca_v_1.2 (L-type calcium channel) in the failing myocardium were increased by T3 and immunoblot analysis further supporting a potential T3 effect on the TT-associated proteins, BIN1 and Jph2.

In conclusion, low dose T3 treatment initiated immediately after myocardial infarction attenuated adverse TT remodeling, improved calcium dynamics and contractility, thus supporting the potential therapeutic utility of T3 treatment in heart failure.

## Introduction

Patients with heart failure (HF) resulting from coronary artery disease or other causes that lead to systolic or diastolic dysfunction often present with thyroid abnormalities with low circulating thyroid hormones (subclinical hypothyroidism or low T3 syndrome), occurring with increasing frequency with increased disease severity (Ascheim and Hryniewicz 2002; Ertugrul et al. 2011; Pingitore et al. 2005; Rothberger et al. 2017; Hayashi et al. 2016; Iervasi et al. 2003; Hamilton et al. 1990; Kannan et al. 2018). A recent Meta-analysis of 35 studies incorporating more than half million participants showed that subclinical hypothyroidism was associated with cardiovascular diseases (CVD) including coronary artery disease, dilated cardiomyopathy, heart failure and atrial fibrillation, and all-cause mortality (Moon et al. 2018). Thyroid hormone replacement therapy of patients with HF or atrial fibrillation has shown to provide significant cardiovascular benefit (Pingitore et al. 2008; Wandell et al. 2017; Moruzzi et al. 1996; Hamilton and Stevenson 1996; Hamilton et al. 1998). Preclinical models of HF have indicated that restoring TH function improves systolic and diastolic function with reduction in myocardial fibrosis and myocyte loss, with attenuation of adverse ventricular remodeling and increased resistance to arrhythmia induction (Rajagopalan et al. 2016; Weltman et al. 2014; Zhang et al. 2018).

In heart failure, transverse tubules (TT) in the remodeled cardiomyocyte appear dilated and decreased in number while longitudinal tubules increase with greater distances between sarcoplasmic reticulum (SR) and TT (Hong and Shaw 2017; Hasenfuss 1998). Advances in super-resolution cell imaging have confirmed these TT changes in heart failure that lead to diminished synchronous and slowed Ca^2+^ release resulting in impaired excitation-contraction coupling and contractile dysfunction (Hong et al. 2014; Hong et al. 2010; Singh et al. 2017; Ibrahim et al. 2012). Adverse remodeling of TT has been documented in many animal models of HF and recently confirmed in failing human hearts (Hong et al. 2012; Schobesberger et al. 2017; Seidel et al. 2017; Pinali et al. 2017). Changes in several proteins that function as scaffolding, folding or bridging proteins between SR and TT have been implicated in TT alterations in HF. These include junctophilin-2 (Jph2) that anchors sarcolemma or junctional SR to the TT, and bridging integrator-1 (BIN1) that promotes folding of TT membranes to create microdomains, and chaperones the delivery of L-type calcium channel (LTCC) via microtubules to these TT domains (Hong et al. 2014; Hong et al. 2012; Caldwell et al. 2014). Jph2 gene therapy in mice with HF was shown to normalize RyR2-induced calcium release, while β-adrenergic receptor blocker treatment improved TT integrity and Jph2 expression in mice after myocardial infarction (Reynolds et al. 2016; Fu et al. 2016; Lyon et al. 2012; Chen et al. 2012).

Thyroid hormones regulate many aspects of cardiac and vascular function, in part by controlling expression of genes regulating myocyte Ca^2+^ handling and contractility (Klein and Ojamaa 2001; Gerdes and Ojamaa 2016; Jabbar et al. 2017). TH deficiency as may occur in HF, was reported to reduce SR Ca^2+^ and decrease the rate of SR Ca^2+^ release with a blunted Ca^2+^ transient amplitude (Montalvo et al. 2018). A recent study showed that TH promoted functional TT development in human-induced pluripotent stem cell-derived cardiomyocytes (Parikh et al. 2017). In engineering heart tissue, supplementation with T3 combined with low-rate contractile activity promoted the structural and functional maturation of cardiomyocytes including increased cross-sectional area, improved twitch force, organized sarcomeres and T-tubules, M-bands and intercalated discs (Jackman et al. 2018). In light of mounting evidence of thyroid dysfunction in HF, and improved cardiomyocyte contractility with TH treatment, we hypothesized that low cardiac tissue T3 may underlie the maladaptive remodeling of TT in failing hearts, and that normalizing thyroid function in this disease setting could be beneficial. Therefore, the purpose of the present study was to investigate whether T3 treatment in a rat model of heart failure could improve cardiac function by attenuating adverse TT remodeling, and thus maintain normal cellular calcium transients and contractile function.

## Materials and methods

### Animal model and treatment protocol

Animals were treated in accordance with the National Institutes of Health *Guidelines for the Use and Care of Laboratory Animals* (HHS Pub. No.85–23), and study protocols were approved by the Institutional Animal Care and Use Committee of the New York Institute of Technology College of Osteopathic Medicine. Female Sprague-Dawley rats (220–250 g) (Envigo RMS, Inc., Indianapolis, IN) underwent a thoracotomy and permanent ligation of the left anterior descending coronary artery (myocardial infarction, MI) or sham surgery without occlusion of the coronary vessel. The morning after surgery, surviving MI animals were randomized to receive triiodo-L-thyronine (T3; SigmaAldrich, St. Louis, MO) or vehicle in their drinking water for 16 weeks. Stock T3 was prepared by dissolving in an ethanol/glycerol solution and then diluted in the drinking water at a concentration calculated to deliver 5μg/kg/d as we have previously published (Rajagopalan et al. 2016). Rats were housed under controlled temperature conditions with 12 h light/dark cycles, and food and water were available ad libitum. Water consumption was recorded twice weekly and body weights once weekly to calculate the amount of stock T3 added to the drinking water to deliver the desired dose. This study was repeated two separate times with animals used either for isolation of ventricular myocytes or for tissue analysis. At 15–20% mortality due to MI surgery, the final number of animals for the isolated cardiomyocyte studies was *n* = 12 for MI + Veh, *n* = 13 for MI + T3, and *n* = 10 for the sham group. Six animals per study group were designated for ventricular RNA and protein analyses.

### Echocardiographic and hemodynamic measurements

Cardiac function was recorded in lightly anesthetized animals (isoflurane at 3% induction, 1.5% maintenance) after the 16 wk. study period as previously described (Zhang et al. 2018). Two-dimensional echocardiograms (GE Vivid 7-Dimension Ultrasound, Horten, Norway) using a M12 L linear array transducer (5–13 MHz) were obtained from LV short- and long-axis views and analyzed in M-mode to measure atrial and ventricular structural and functional parameters in systolic and diastolic phases. Immediately following echocardiographic recordings, LV hemodynamics were recorded by catheterization using a 1.9F pressure-volume catheter (Transonic SciSense, Canada) advanced through the right carotid artery into the LV to record heart rate (HR), LV end systolic and diastolic pressures (LVESP, LVEDP), maximum LV pressure developed and changes in pressures over time (±dP/dt). Data were recorded for 20 min. until hemodynamic parameters reached steady state, and the final ~ 100 cardiac cycles were used for calculations. Data were acquired and analyzed using LabScribe software (iWorx Systems, Dover, NH).

### Serum thyroid hormone assays

Following cardiac functional recordings on closed-chest animals, a left thoracotomy exposed the heart and blood was obtained from the right ventricle. Serum was collected, aliquoted and stored at − 20 °C until it was analyzed for free T3, total T3, total T4 and TSH using enzyme-linked immunosorbent assay kits (Monobind Inc., Lake Forest, CA) following the manufacturer’s recommended protocol.

### Left ventricular myocyte isolation

After blood was collected, the aorta was clamped ~ 5 mm above its entry into the heart, and a solution containing 5 mM EDTA was injected slowly into the right and left ventricles to flush the coronary vessels. Briefly, as previously published (Huang et al. 2007), the heart was removed and the aorta was tied to a cannula on a perfusion apparatus (Radnoti LLC, Covina, CA) allowing retrograde perfusion of the heart with a modified calcium-free Krebs-Henseleit buffer (KHB) containing collagenase (0.18 g/50 ml KHB; Worthington Biochemical Corp., Lakewood, NJ) that was warmed to 37 °C. After ~ 45 min. of digestion, the perfusion was stopped, the atria and right ventricle were removed and the left ventricle was cut into small pieces and gently teased apart to isolate cardiomyocytes. All isolated LV myocytes were combined without separation of those located proximal or distal to the infarct zone. Enzymatic digestion was terminated with EDTA-containing KHB and cells collected by centrifugation. Following calcium reintroduction, the myocytes were maintained in Tyrode’s buffer for immediate IonOptix analysis or plated onto laminin-coated coverslips or glass-bottom 35 mm dishes in medium M199 containing 10% FBS and 2,3-butanedione monoxime (BDM, 10 mM). Ventricular myocytes were allowed to adhere for 2 h prior to live cell staining for T-tubule/sarcolemma imaging. An aliquot of cardiomyocytes derived from each heart was fixed in 4% PFA for subsequent measurement of sarcomere and cell lengths using bright field images on an Olympus BX53 microscope at 40x power.

### Calcium transient and contractile recordings

The IonOptix Imaging System allows simultaneous recordings of cardiomyocyte contractile cycles and intracellular calcium transients in response to electrical field stimulation using video-based edge detection of sarcomere length changes coupled to capture of fluorescence signals. The ratio metric dye, Fura2, allows direct measure of intracellular free calcium by capturing its fluorescence signals emitted at excitation wavelengths of 340 and 380 nm and expressed as fluorescence ratio at 340/380 nm. Lyophilized Fura2/AM (Molecular Probes, Invitrogen) was reconstituted in DMSO to a stock concentration of 1 mM, aliquoted for single use and stored at − 20 °C. Isolated ventricular myocytes incubated in Tyrode’s buffer containing 1.8 mM Ca^2+^ were loaded with Fura2/AM (1uM final) for 5 min. at room temperature, then thoroughly washed and placed in a perfusion chamber mounted on a Motic® AE31 inverted microscope and visualized at 40X magnification. Criteria for inclusion of myocytes for analysis: rod shaped with defined cell edges, no blebs or cauliflower-shaped cell ends, quiescent when unstimulated, and stable contractility when stimulated for ~ 5 min. Myocytes were field stimulated at 1 Hz (4 V) and mechanical properties were recorded at a sampling rate of 240 Hz. After cell contractions reached steady state, contractile cycles and calcium transients of each cell were analyzed using the IonWizard™ v6.5 acquisition software. Data from ~ 20 cycles per cell and ~ 25 cells per heart were used to calculate average values of each parameter recorded. Indices for isotonic shortening and re-lengthening are listed in Table 2 and calcium transients in Table 3.

### Confocal imaging of T-tubules

Live cardiomyocytes adhered to laminin-coated No.0 glass bottom microwells of 35 mm culture dishes (MatTek Corp., Ashland, MA) were washed in HBSS (with calcium and magnesium chloride; Gibco, Life Technologies) containing 10 mM BDM to remove serum. Cells were then incubated with di-8-ANEPPS (5 uM in HBSS/BDM; Biotium Inc., Fremont, CA) for 20 min. at 37 °C as published by others (Hong et al. 2014; Singh et al. 2017; Schobesberger et al. 2017; Caldwell et al. 2014; Reynolds et al. 2016). Cells were washed and immediately imaged with a 63x objective under oil immersion using a Leica confocal microscope (DMI6000 SP5) housed in an enclosed unit heated to 37 °C. Di-8-ANEPPS was excited at 488 nm and emission wavelengths > 505 nm were collected. Images from ~ 8 z-stacks (0.6 nm z-step) per cell were captured (~ 10 cells/heart) and subsequently analyzed for TT periodicity using an algorithm developed by Pasqualin et al. (Pasqualin et al. 2015) to calculate the transverse organization level of the T-tubule system based on the peak amplitude in the Fourier spectrum of the image at the TT frequency, or TT power. The higher the TT power value, the greater the TT periodicity or organization. This TTorg analytical software is freely available as a plug-in on ImageJ. An additional analytical program, AutoTT designed by Guo and Song (Guo and Song 2014) was made available to the authors. This analysis allows simultaneous measurement of the longitudinally (LE)-oriented and transversely (TE)-oriented T-tubules in each of the ANEPPS-labeled cardiomyocytes, and outputs the TT densities as percent total number of pixels of skeletalized TE or LE divided by the total number of pixels of the region of interest (ROI).

A second approach was used to assess T-tubule organization that involved labeling of plasma membrane glycoproteins using Alexa 488-conjugated wheat germ agglutinin (WGA-488; Molecular Probes). Live cardiomyocytes adhered to laminin-coated glass coverslips were incubated with WGA-488 (5 μg/ml HBSS/BDM) for 20 min. at 37 °C, after which unbound WGA-488 was removed by thorough washes with HBSS/BDM. Cells were then fixed with 4% paraformaldehyde (PFA) for 15 min. at room temperature. Cells were washed of PFA and the coverslips mounted onto glass slides in Prolong™ Gold antifade reagent with DAPI (Invitrogen). Images were captured using a Nikon C2 LSC microscope with 60x objective under oil immersion with a numerical aperture (NA) of 1.35 and ~ 0.14 um/pixel. Ten to fifteen z-stacks (at 0.4 um z-steps) were captured for each cell and 15–20 cells per heart were analyzed for TT periodicity using the TTorg software program.

### Real-time quantitative PCR

Frozen left ventricular tissues were pulverized and ~ 50 mg samples were homogenized in QIAzol lysis reagent using a motor-driven Teflon pestle and glass homogenizer followed by brief 2 s bursts of a Polytron (Fisher Scientific). Total RNA was extracted using RNeasy Mini spin columns according to the manufacturer’s instructions (Qiagen, Germantown, MD) and RNA concentration was measured by absorbance at 260 nm using Nanodrop 1000 (ThermoFisher Scientific). cDNA was reverse transcribed from 1 μg RNA using anchored-oligo(dT)_18_ and random hexamer primers (Transcriptor First Strand cDNA Synthesis Kit, Roche Diagnostics Corp., Indianapolis, IN). Real-time PCR (StepOnePlus, Applied Biosystems ThermoFisher) using SYBR Green technology (RT^2^ SYBR Green ROX qPCR) to amplify BIN1, Jph2, RyR2, Ca_v_1.2 (LTCC), SERCA2, PLN, α-MHC and β-MHC cDNAs with specific primers designed and verified by Qiagen (Valencia, CA). Large ribosomal protein, Rplp1, or glyceraldehyde-3-phosphate dehydrogenase (GAPDH) was used as the housekeeping gene for normalization of amplified PCR products in each reaction. The threshold cycle values were used for ΔΔCt calculations of PCR cycle amplifications to determine mRNA content relative to the sham control samples.

### Immunoblot analysis

Pulverized frozen LV tissue (200 mg) was homogenized in a HEPES buffer (containing 200 mM sucrose, 0.4 mM CaCl2, 20 mM HEPES pH 7.4, protease-phosphatase inhibitors), with modification of a published protocol (Hong et al. 2012). Briefly, tissue homogenates were centrifuged at low-speed (1400×g) and the supernatants collected and centrifuged at 41,000×g to pellet the microsomal fractions. The microsomes were resuspended in 30 mM imidazole pH 6.8 buffer (containing 100 mM NaCl, 8% sucrose, protease-phosphatase inhibitor cocktail), layered over a sucrose cushion and centrifuged at 150,000×g for 16 h at 4 °C. Protein concentration was determined in the pelleted microsomes and 30 μg protein/sample were loaded on 4–20% gradient gels (Mini-Protean® TGX™, BioRad Inc., Hercules, CA) for SDS-PAGE (immunoblots for RyR and Ca_v_1.2 detection) or 10% SDS-polyacrylamide gels (for BIN1 and Jph2 detection). Proteins were transferred to nitrocellulose membranes that were then stained with Ponceau Red solution (SigmaAlrich, St. Louis, MO) to correct for gel loading variations. Membranes were blocked with TBST/5% milk and incubated overnight at 4 °C with the following primary antibodies (all at 1:500 dilution): rabbit polyclonal anti-Ca_v_1.2 antibody (cat. no. ACC-003; Alomone Labs, Jerusalem, Israel), mouse monoclonal (mAb) anti-RyR antibody (cat. no. MA3–916/C3–33; Invitrogen, ThermoFisher), mouse mAb anti-BIN1 (cat no.200–301-E63; Rockland Inc., Limerick PA), mouse mAb anti-Jph2 (cat no.600–401-CC5; Rockland Inc.). Secondary antibodies used were horseradish peroxidase (HRP)-conjugated goat anti-rabbit or anti-mouse IgG(H + L) (1:10,000; Invitrogen). HRP was detected using chemiluminescence reagent (Pierce SuperSignal™ West Pico or Femto Substrate; ThermoFisher) and visualized using Amersham Imager 600. Specific protein band signal intensity was measured using ImageJ densitometry analysis software. Ponceau Red stained bands closest in molecular weight to the proteins of interest were used to normalize for gel loading.

### Statistical analysis

Data were assessed for normality distribution and equal variance, and are presented as mean ± SD. Statistical significance among groups was determined by one-way ANOVA followed by Tukey’s multiple-group comparisons. Data that did not pass normality distribution were analyzed for significance using Kruskal-Wallis non-parametric test followed by Dunn’s multiple group comparisons using GraphPad Prism v7.0 statistical software (GraphPad Software, Inc., San Diego, CA). A *p*-value < 0.05 was considered statistically significant.

## Results

### Serum thyroid hormones

**Fig. 1:**
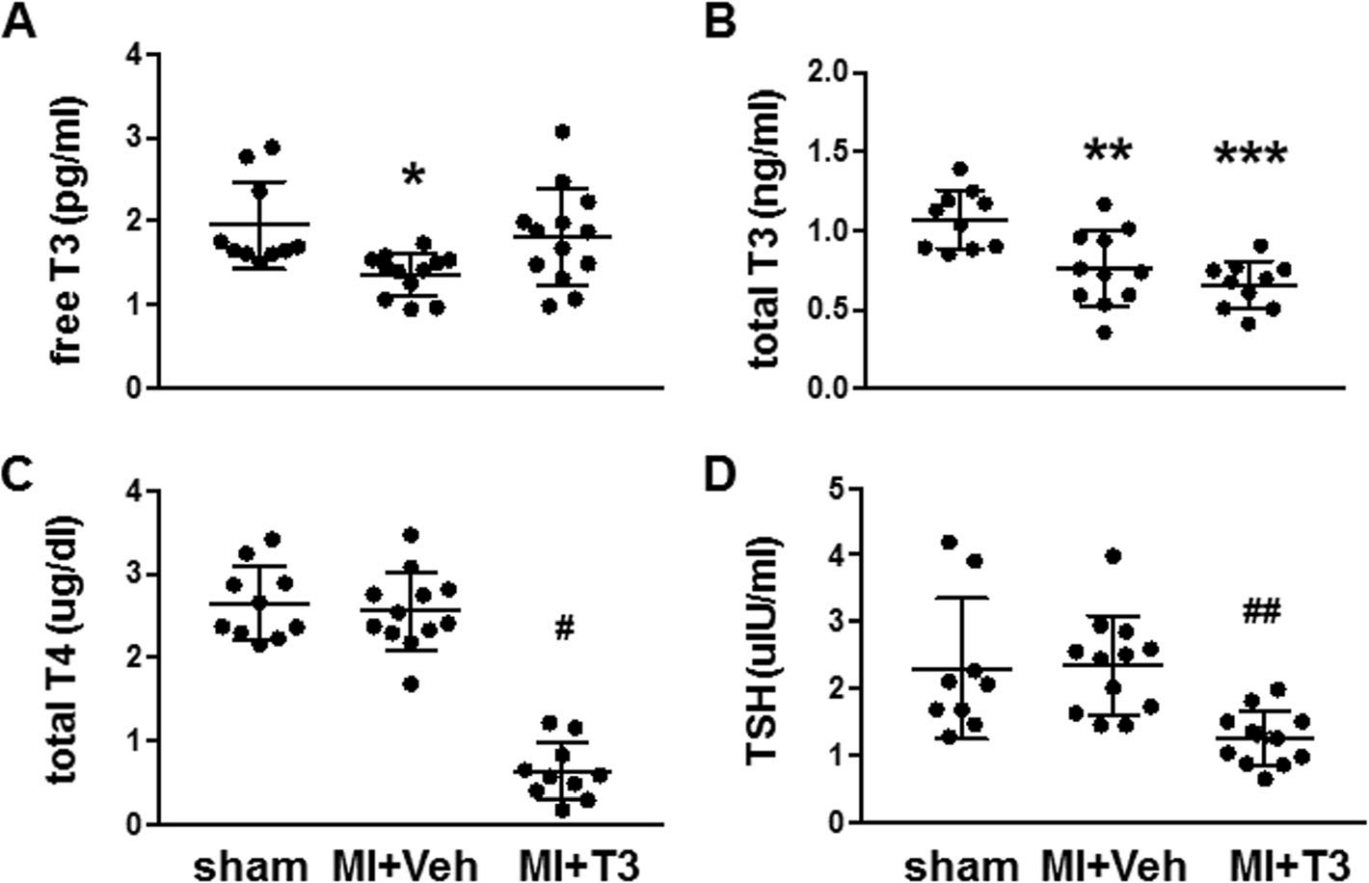
Serum thyroid hormone concentrations. **a** free triiodo-L-thyronine (T3); **b** total T3; **c** total L-thyroxine (T4); **d** thyrotropin/thyroid stimulating hormone (TSH). Concentrations are indicated. Scatter plots represent individual rat hormone values, with lines indicating mean ± SD per group. *n* = 10–13 rats/group. One-way ANOVA followed by Tukey’s test of multiple group comparisons. *p < 0.05 vs sham; **p < 0.01 vs sham; ***p < 0.001 vs sham; ^#^*p* < 0.001 vs sham and MI + Veh; ^##^*p* < 0.01 vs sham and MI + Veh. MI, myocardial infarction; Veh, vehicle

Total and free T3 concentrations in serum were significantly reduced while total T4 was unchanged in the MI + Veh group suggesting low T3 syndrome as often observed in heart failure (Ascheim and Hryniewicz 2002; Iervasi et al. 2003) (Fig. 1a-c). Treatment of MI rats with T3 at a low dose of 5 μg/kg for 16 weeks normalized free T3, and as expected suppressed the hypothalamus-pituitary-thyroid axis resulting in low serum TSH and total T4 (Fig. 1c,d). These results indicate thyroid dysfunction or low T3 syndrome in the untreated MI model, and that the low T3 dose provided effective hormone replacement without producing hyperthyroidism.

### Echocardiographic data of left ventricular (LV) function

At the end of the study, body weights of sham, MI + Veh and MI + T3 groups were not significantly different at 288 ± 14, 284 ± 19 and 304 ± 24 g, respectively. Heart rates as recorded by echocardiography showed no differences among the groups at 300 ± 31, 304 ± 26 and 347 ± 77 bpm, respectively (Table 1). Compared to sham animals, LV end systolic and diastolic volumes were increased significantly in the MI rats, and these values were returned towards sham values with T3 treatment, resulting in a significantly (*p* < 0.05) increased ejection fraction of 50 ± 5% vs 43 ± 8%, MI + T3 vs MI + Veh, respectively. As would be expected, anterior wall thickness was decreased in MI compared to sham animals, but wall thickness in systole was significantly greater in MI + T3 compared to MI + Veh suggesting improved ventricular remodeling with T3 treatment. The impaired LV function with increased filling pressures in MI + Veh animals resulted in a 40% increase in left atrial diameter that was decreased by T3 treatment to an atrial chamber size not different from sham animals (Table 1).

**Table 1:** Left ventricular echocardiographic measurements

	Sham	MI + Veh	MI + T3
Heart Rate (bpm)	300 ± 31	304 ± 26	348 ± 77
Ejection Fraction (%)	82 ± 4	43 ± 8*	50 ± 5*#
Left Atrial Diameter (mm)	3.70 ± 0.49	5.21 ± 1.22*	4.68 ± 1.69
LV End Systolic Volume (cm^3^)	0.15 ± 0.05	1.42 ± 0.39*	1.17 ± 0.36*
LV End Diastolic Volume (cm^3^)	0.83 ± 0.18	2.44 ± 0.38*	2.32 ± 0.63*
Anterior wall thickness systole (mm)	2.00 ± 0.22	0.84 ± 0.17*	1.12 ± 0.33*#
Anterior wall thickness diastole (mm)	1.16 ± 0.07	0.76 ± 0.17*	0.88 ± 0.19*
Posterior wall thickness systole (mm)	2.08 ± 0.19	2.05 ± 0.13	2.11 ± 0.11
Posterior wall thickness diastole (mm)	1.15 ± 0.06	1.17 ± 0.05	1.20 ± 0.07

Values are mean ± SD. MI, myocardial infarction; Veh, vehicle; T3, triiodothyronine. Sham, *n* = 11; MI + Veh, n = 11; MI + T3, *n* = 10. One-way ANOVA, post-hoc Tukey’s multiple group comparisons. *p < 0.05 vs sham, #*p* < 0.05 vs MI + Veh

### In vivo hemodynamic measurements

**Fig. 2:**
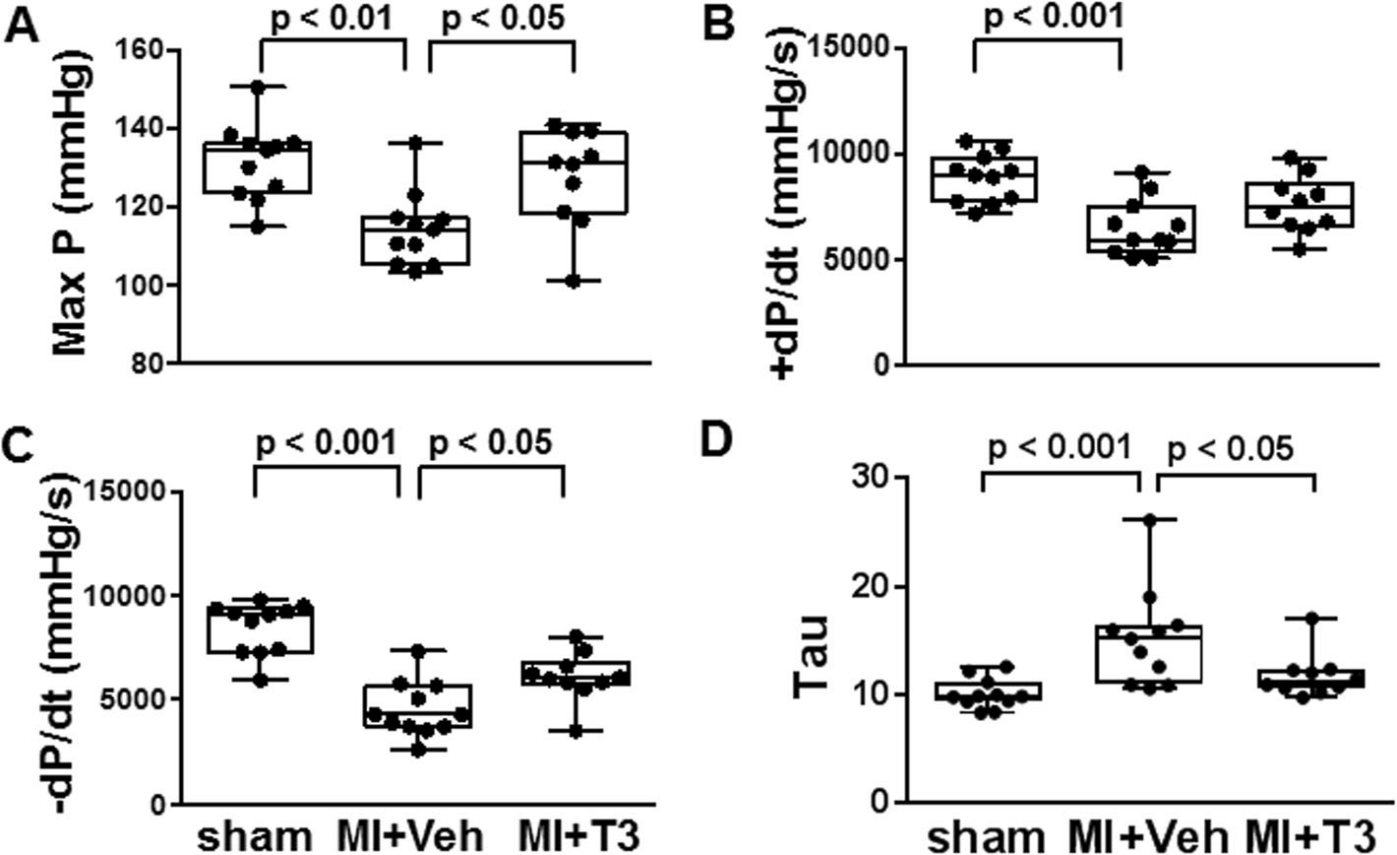
Left ventricular pressure measurements. Box-and-whisker plots indicate median value, box at 25th/75th percentile, and whiskers are max/min values; dots represent values from individual animals. **a** Max P, maximum developed LV pressure; first derivative of LV pressure curve during systole (**b**) + dP/dt, and diastole (**c**) –dP/dt; (**d**) Tau, exponential decay time constant in diastole. Statistical analysis by one-way ANOVA with Tukey post hoc multi-group analysis; *p*-values between groups are indicated

Left ventricular pressure measurements recorded in lightly anesthetized animals showed significant decreases in the maximum LV pressure development (Max P) in the MI + Veh rats compared to sham (*p* < 0.01) and complete normalization with T3 treatment (Fig. 2a). Rate of pressure development during systole (+dP/dt) was decreased in MI + Veh compared to sham animals (*p* < 0.001) and returned towards normal with T3 treatment (Fig. 2b). During diastole, the rate of relaxation (−dP/dt) and the isovolumic relaxation time constant, Tau, showed significant impairment in MI + Veh and these measures were significantly improved with T3 treatment (Fig. 2c,d).

### Isolated ventricular myocyte contractility and calcium transients

**Fig. 3:**
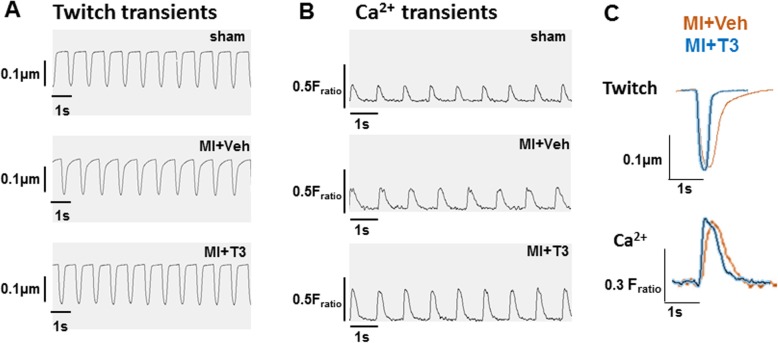
Recordings of representative calcium and contractile/twitch transients. Simultaneous recordings of calcium and contractile transients of field stimulated (1 Hz) single cardiomyocytes captured by the IonOptix imaging system: **a** displacement (Twitch) transients over time of representative single cardiomyocytes from sham, MI + Veh and MI + T3 rats. Vertical scale bar = 0.1 um sarcomere length, horizontal scale bar = 1 s; **b** Ca^2+^ transients of single myocytes from each study group showing ratio of fluorescence intensities excited at 340 and 380 nm (F_ratio_ = vertical scale bar); **c** overlay of twitch and Ca^2+^ transients from representative cardiomyocytes isolated from MI + Veh and MI + T3 rats

Freshly isolated ventricular myocytes that did not elicit spontaneous contraction and did not have irregular membranes or blebs upon microscopic inspection were used to record sarcomere shortening by video-based edge detection during field stimulation at 1 Hz. Calcium transients were recorded simultaneously from these myocytes preloaded with Fura2/AM. Upon electrical stimulation, maximum shortening (contraction) velocity was decreased in cardiomyocytes isolated from MI + Veh rats compared to sham animals, such that the time at peak shortening was significantly longer (0.197 ± 0.011 vs 0.165 ± 0.024 s, MI + Veh vs sham, respectively; *p* < 0.05) (Table 2). T3 treatment increased maximum shortening velocity, thereby significantly decreasing the time at peak shortening (0.161 ± 0.021 vs 0.197 ± 0.011 s, MI + T3 vs MI + Veh, respectively) (Table 2). More striking effects were observed during the relaxation (or re-lengthening) phase of the contractile cycle. Maximum velocity of relengthening was reduced in MI + Veh myocytes, and significantly increased to sham values with T3 treatment (1.593 ± 0.325, 2.984 ± 1.410 and 2.299 ± 0.837 um/sec, MI + Veh, MI + T3 and sham, respectively). All measures of re-lengthening were significantly slower and prolonged in the MI + Veh myocytes compared to sham, and these values were significantly faster and occurred in a shorter time period with T3 treatment (Table 2). The exponential decay time constant, Tau, that characterizes the speed of relengthening, and the area under the curve during the re-lengthening phase of the contractile cycle, all indicated a positive effect of T3 treatment on contractile dynamics of myocytes after MI (Table 2). Representative single myocyte twitch recordings from each group of animals, and a composite showing an overlay of traces from MI + Veh and MI + T3 twitches are shown in Fig. 3a and c.

**Table 2:** Isolated cardiomyocyte contractile measurements

		Sham	MI + Veh	MI + T3
Maximum Shortening Velocity (μm/s)		3.513 ± 0.808	3.125 ± 0.726	3.703 ± 1.213
Maximum Relengthening Velocity (μm/s)		2.299 ± 0.837	1.593 ± 0.325	2.984 ± 1.410#
Time at Maximum Relengthening Velocity (s)		0.248 ± 0.048	0.314 ± 0.019*	0.233 ± 0.037#
Peak Height as Percent of Baseline		11.17 ± 1.97	11.70 ± 1.68	12.11 ± 2.96
Time at % from Baseline to Peak Shortening (s)				
	50%	0.049 ± 0.006	0.053 ± 0.005	0.054 ± 0.009
	90%	0.109 ± 0.014	0.130 ± 0.006	0.105 ± 0.020#
Time at Peak Shortening (s)		0.165 ± 0.024	0.197 ± 0.011*	0.161 ± 0.021#
Time at % from Peak Shortening to Full Relengthening (s)				
	50%	0.258 ± 0.050	0.326 ± 0.018*	0.261 ± 0.071#
	90%	0.353 ± 0.071	0.443 ± 0.024*	0.324 ± 0.071#
Tau (exponential decay time constant) (s)		0.048 ± 0.019	0.067 ± 0.008	0.045 ± 0.021#
Area Under Curve of Relengthening Phase		0.021 ± 0.006	0.029 ± 0.004*	0.020 ± 0.005#

Values are mean ± SD. MI, myocardial infarction; Veh, vehicle; T3, triiodothyronine. Tau is exponential decay time constant of the sarcomere relengthening phase. Sham, *n* = 7; MI + Veh, *n* = 8, MI + T3, *n* = 8. One-way ANOVA, multiple group comparisons using Tukey’s analysis. *p < 0.05 vs Sham, #*p* < 0.05 vs MI + Veh

Calcium transients were recorded simultaneously with twitch recordings, and representative traces of cells from each study group are shown in Fig. 3b. Calcium transients are presented as a ratio of fluorescence intensity at 340 nm/380 nm (F_ratio_). In Fig. 3c the overlay of calcium transients from MI + T3 and MI + Veh myocytes shows that compared to vehicle treated MI rats, T3 treatment shifted the Ca^2+^ transient to the left, indicating overall faster kinetics. The values for maximum rates of rise and fall of the Ca^2+^ transient are reported in Table 3. Because Ca^2+^ peak heights were different among cardiomyocytes, max rates of Ca^2+^ rise and fall were normalized to their peak heights. Although not statistically significant, the rate of Ca^2+^ rise was faster but occurred later in the MI cardiomyocytes compared to either sham or MI + T3 myocytes, suggesting that SR Ca^2+^ release was delayed in MI cells potentially due to decreased coupling of LTCC to RyR and thus slowing of Ca^2+^-induced Ca^2+^ release. Additionally, the rate of Ca^2+^ fall was faster and occurred sooner in the T3-treated cardiomyocytes (5.38 ± 0.61 vs 4.83 ± 0.24, MI + T3 vs MI + Veh, respectively) reflecting more efficient Ca^2+^ removal from the cytoplasm into the sarco (endo) plasmic reticulum by the SR calcium-ATPase (SERCA2), a known T3-responsive gene. These results can be further characterized by measuring the area under the curve from peak cytosolic Ca^2+^ to baseline (complete SR re-uptake) which was smaller in the T3 vs vehicle treated MI cardiomyocytes (0.050 ± 0.014 vs 0.072 ± 0.019). Taken together, the twitch/contractile and calcium transient data support a positive effect of T3 on cytosolic Ca^2+^ re-uptake, and potentially an effect on SR Ca^2+^ release, resulting in more rapid myocyte contraction (shortening) and relaxation (relengthening) velocities.

**Table 3:** Cardiomyocyte calcium transient measurements (rise and fall)

	Sham	MI + Veh	MI + T3
Ca^2+^ Rise (Max Rate/Peak Ca^2+^ Height)	67.1 ± 4.1	72.2 ± 7.3	67.0 ± 9.8
Time at max rate of Ca^2+^ Rise (s)	0.016 ± 0.002	0.017 ± 0.003	0.015 ± 0.002
Ca^2+^ Fall (Max Rate/Peak Ca^2+^ Height)	4.84 ± 0.25	4.83 ± 0.24	5.38 ± 0.61
Time at max rate of Ca^2+^ Fall (s)	0.175 ± 0.001	0.183 ± 0.008	0.171 ± 0.016
Area Under Curve of Ca^2+^ Fall Phase	0.068 ± 0.013	0.072 ± 0.019	0.050 ± 0.014

Values are mean ± SD. MI, myocardial infarction; Veh, vehicle; T3, triiodothyronine. Sham, *n* = 5; MI + Veh, n = 7, MI + T3, n = 5; Max Rate is max velocity of Ca^2+^ rise (release) or Ca^2+^ fall (re-uptake) phase of the transient

### T3 increases T-tubule periodicity in post-MI cardiomyocytes

**Fig. 4:**
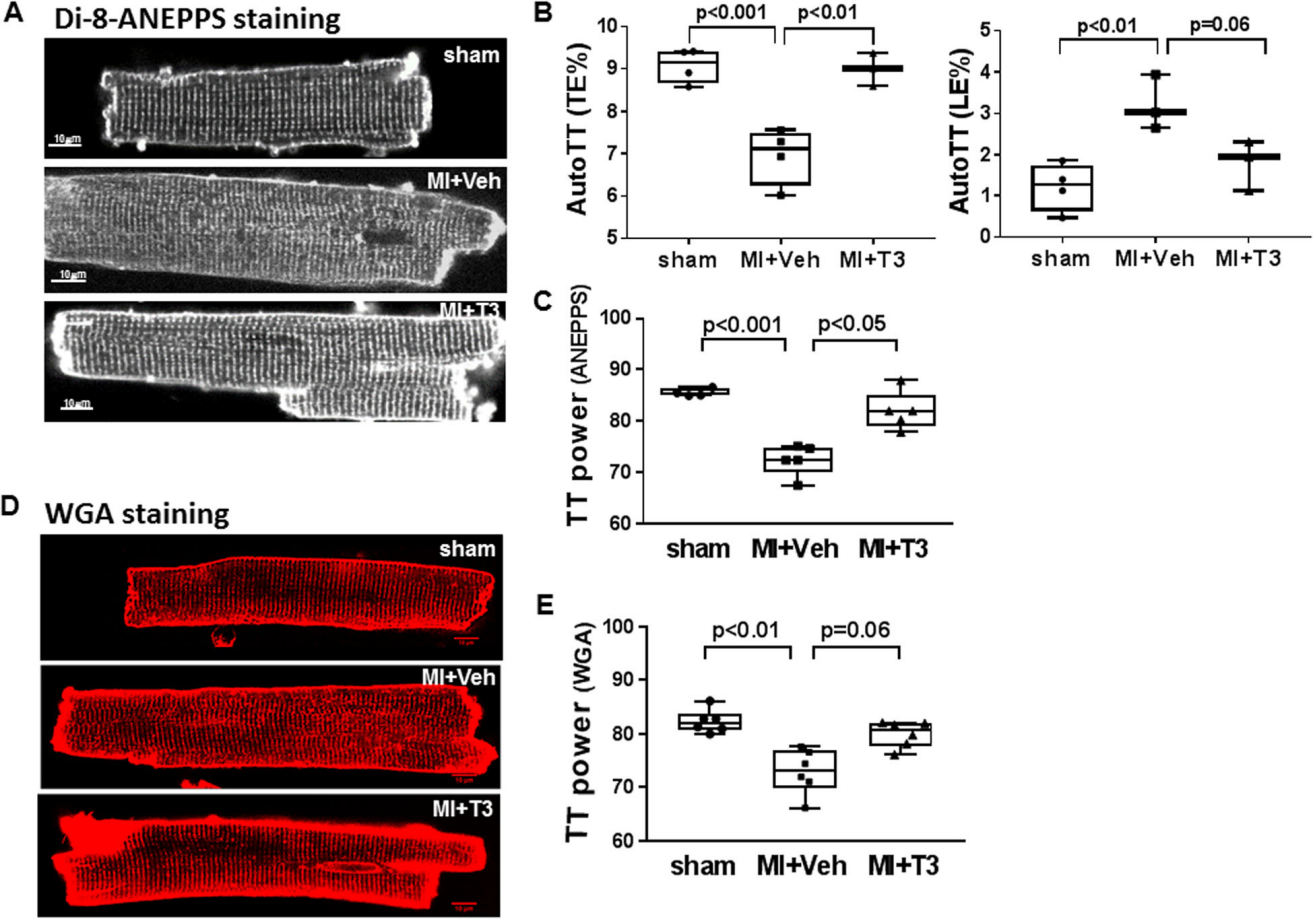
Confocal imaging of cardiomyocyte T-tubule networks. **a** Representative images of cardiomyocytes from each study group labeled with di-8-ANEPPS captured on a Leica LSM using a 63x objective. Scale bar = 10 um. **b** AutoTT quantitative analysis of di-8-ANEPPS images showing tubule densities as percent total number of pixels of skeletalized transverse-oriented tubules (TE%) or longitudinal-oriented tubules (LE%) divided by the total number of pixels of the region of interest (ROI). Each dot represents averaged values from one animal. Box-and-whisker plots show median value, box fences at 25th /75th percentiles, and whiskers at max/min values. **c** TT power analysis of di-8-ANEPPS images. Each dot is the average TT power value of 6–8 z-stacks captured per cell of ~ 10 cells per heart. Box-and-whisker plots as described in (**b**); *n* = 4–5 rats/group. **d** Representative images of cardiomyocytes labeled with AlexaFluor-488 conjugated wheat germ agglutinin (WGA-488) and images captured using Nikon C2 LSC microscope with 63x oil immersion objective. Scale bar, 10 um. **e** TT power quantitative analysis of WGA images. Each dot is the average TT power value of ~ 12 z-stacks captured per cell of 15–20 cells per heart. Box-and-whisker plots as described above; *n* = 6–7 rats/group. One-way ANOVA, with *post-hoc* Tukey analysis, *p*-values between groups are indicated

T-tubules facilitate the juxtaposition of sarcolemmal LTCCs to junctional SR ryanodine receptors (calcium-release channels) that couple extracellular calcium entry with SR calcium release. Since the in vivo hemodynamic data and single cell twitch and Ca^2+^ transients indicated that T3 treatment of MI rats improved these processes, we assessed whether T3 treatment altered T-tubule structure in the failing hearts. T-tubules were visualized using di-8-ANEPPS or WGA-488 staining and representative confocal images of cardiomyocytes from each study group are shown in Fig. 4a & d. AutoTT analysis of ANEPPS-stained cells showed significant reductions of transversely-oriented tubules (TE%) in failing hearts while longitudinally-oriented tubules were increased significantly (LE%) (Fig. 4b). T3 treatment attenuated these adverse T-tubule changes. Furthermore, TTorg analysis of both ANEPPS-stained cardiomyocytes (Fig. 4c) and WGA-stained cardiomyocytes (Fig. 4e) showed that the periodicity of the transverse-oriented tubules (calculated as TTpower) was significantly disrupted in MI hearts compared with sham, and that T3 treatment significantly increased the TT regularity .

### T-tubule and SR associated proteins and RNA expression

**Fig. 5:**
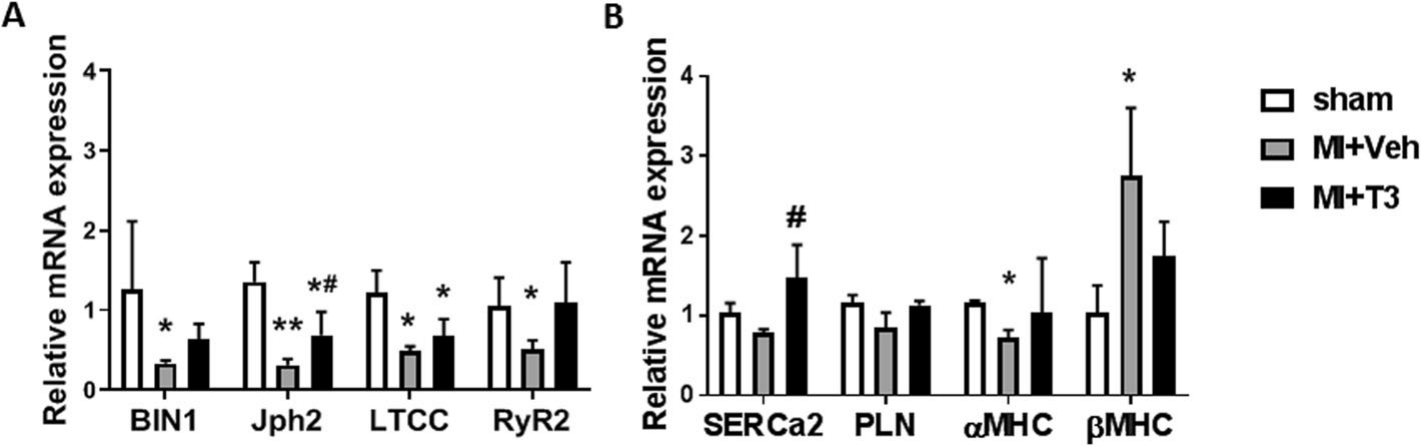
RNA expression of T-tubule and SR genes RT-qPCR analysis of LV tissue for mRNA content of (**a**) bridging integrator-1, BIN1; junctophilin 2, Jph2; L-type calcium channel, LTCC; ryanodine 2, RyR2 and (**b**) SR calcium ATPase, SERCa2; phospholamban, PLN; alpha- and beta-myosin heavy chain, αMHC and βMHC. The binary log of ΔΔCt calculations of PCR cycle amplifications was used to determine the fold-change relative to the mean values of the Sham group. Bar graph shows mean ± SD for n = 4–6 hearts/group. Statistical analysis used one-way ANOVA with *post-hoc* Tukey’s multi-group comparisons: *p < 0.05 vs sham; **p < 0.001 vs sham; #*p* < 0.01 vs MI + Veh.

Whether genes encoding T-tubule proteins including BIN1 and Jph2 are T3-responsive has not been reported. Using quantitative RT-PCR, we showed that BIN1 (*p* < 0.01) and Jph2 mRNAs were significantly decreased in MI compared to sham hearts, and that expression of these genes were increased toward sham levels with T3 treatment (Fig. 5a). Similarly, LTCC and RyR2 mRNAs were decreased significantly in MI compared to sham hearts, and a trend upwards with T3 treatment. Whether T3 regulates transcription of these key calcium channels and T-tubule associated proteins by directly activating T3 response elements (TRE) located within the promoter/enhancer regions of these genes will require studies focused on identification of these DNA elements. In addition to these genes expressing TT associated proteins and the LTCC-RyR dyad, we also measured expression of SERCA2 and PLN, key proteins regulating calcium uptake into the SR during diastole. SERCA2 is a known T3-responsive gene and its expression was increased significantly with T3 treatment compared to untreated MI hearts (Fig. 5b). PLN expression did not show any significant changes within the failing myocardium or with T3 treatment. The genes encoding the primary contractile proteins of the heart, the alpha-myosin heavy chain (fast contracting αMHC) and beta-MHC (slow contracting βMHC) are regulated by thyroid hormone (van Rooij et al. 2007; van Rooij et al. 2009) . Their altered expression in MI hearts with a significant decrease in αMHC with reciprocal increase in βMHC are characteristic of a heart failure phenotype (Fig. 5b). T3 treatment normalized expression of these genes, thus explaining in part the improvements measured in contractility and hemodynamics (Fig. 2, Table 2).

**Fig. 6:**
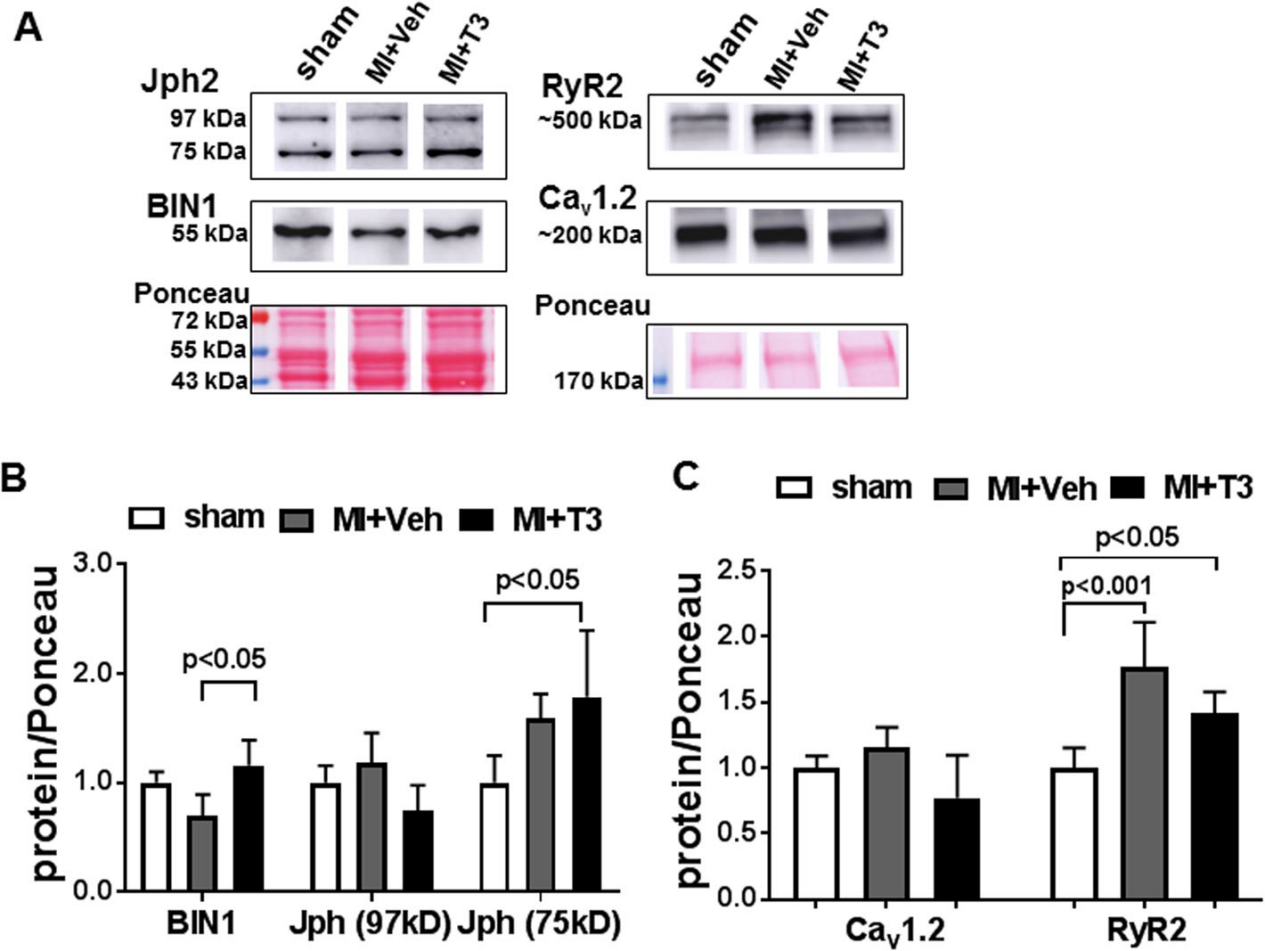
Immunoblot analysis of T-tubule and SR proteins. **a** Representative immunoblots of microsomal fractions from LV tissues of each study group that were probed with anti-BIN1, Jph2, Ca_v_1.2 and RyR2 antibodies. Observed molecular weights (kDa) of each protein are indicated. Ponceau red staining of the immunoblots are shown with molecular weight markers. **b**,**c** Bar graphs show the quantitation of the luminescence intensity of the indicated protein bands, normalized to Ponceau red staining and expressed relative to the mean sham values. Values are mean ± SD for n = 4–6 hearts/group. Statistical analysis using one-way ANOVA with *post-hoc* Tukey’s multiple group comparisons; *p-*values are indicated

Immunoblot analysis of proteins isolated from the microsomal fraction of LV homogenates are represented in Fig. 6a. Quantitation of these proteins involved normalization to Ponceau red staining of protein bands closest in molecular weight to the proteins of interest as shown. Results showed that BIN1 was reduced in MI + Veh and increased significantly in MI + T3 hearts to that measured in sham rats (Fig. 6b). Two Jph2 protein bands were observed: 97 kDa full length protein and 75 kDa fragment, recently identified as a calpain cleaved product that is increased in stressed hearts (Guo et al. 2015; Guo et al. 2018). Quantitation showed that full length 97 kDa protein was unchanged among treatments groups, while the 75 kDa protein was increased in the MI and MI + T3 hearts supporting its reported role as a stress-adaptive transcriptional regulator (Guo et al. 2018) (Fig. 6b). Ca_v_1.2 content in the microsomal fraction did not change in any of the study groups, whereas RyR2 increased significantly in both MI + Veh and MI + T3 hearts compared to sham hearts (Fig. 6c).

## Discussion

The primary findings of this study are that low dose T3 treatment in a rat model of heart failure (HF) prevented adverse remodeling of the cardiomyocyte T-tubule network (TT), improved myocyte contractile kinetics, and normalized calcium transients. Secondly, the molecular mechanisms underlying these responses may in part involve T3-regulated gene expression of TT proteins including BIN1 and Jph2, and their role in maintaining the TT structure.

The permanent artery ligation model of heart failure used in this study remodeled the cardiomyocyte similar to that measured in patients with ischemic cardiomyopathy with increased cell length of ~ 30% compared to non-failing donor hearts without changes in sarcomere length (Gerdes et al. 1992). Echocardiographic measurements of altered chamber dimensions, and significantly decreased measurements of LV pressure development indicated a failing phenotype 16 weeks after MI. This model supported our previously published studies in which serum T3 levels were decreased significantly in heart failure, and in which low dose T3 treatment restored serum T3 to normal without untoward effects (Rajagopalan et al. 2016; Zhang et al. 2018; Weltman et al. 2015). Early studies of compensated cardiac hypertrophy had shown that myocyte surface-to-volume ratio was maintained by greater proliferation of T-tubules than sarcolemma, and that imbalances in growth of these organelles could cause abnormalities in calcium transients and thus contractile dysfunction in HF (Page and McCallister 1973). Unknown was whether the adverse changes in cellular ultrastructure including the T-tubule network in the failing heart could be attenuated by T3 treatment.

In the present study, confocal microscopy of isolated cardiomyocytes stained with WGA or di-8-ANEPPS to visualize T-tubules showed significant reduction in the periodicity of these structures similar to that described by others in animal and human failing myocytes (Singh et al. 2017; Ibrahim et al. 2012; Hong et al. 2012; Schobesberger et al. 2017; Pinali et al. 2017). Novel to this study was the observation that T3 treatment commenced immediately after MI prevented these T-tubule changes or altered the course of this remodeling. Chronic increases in hemodynamic workload after myocardium loss due to infarction or alternatively, due to pressure or volume overload, triggers ventricular remodeling. Frisk et al. showed that T-tubule structures were maximally disrupted in cardiomyocytes proximal to the infarct zone where wall stress was greatest (Frisk et al. 2016). Similarly, Ibrahim et al. showed that mechanical unloading reversed T-tubule remodeling in a rat MI model of heart failure further supporting a role for wall stress in this process (Ibrahim et al. 2012). These studies and others have reported 30 to 45% decreases in T-tubule densities in remodeled cardiomyocytes of various heart failure animal models (Caldwell et al. 2014; Lyon et al. 2012; Chen et al. 2012; Crocini et al. 2016). We isolated myocytes from the entire non-infarcted LV regardless of distance from the infarct area, and found a significant 25% decrease in transverse-oriented tubule density and a two-fold increase in longitudinal-oriented tubule density. Recent reports have shown an absolute requirement for T3 and low-intensity contractile activity in promoting the maturation of T-tubules in engineered cardiac tissues and stem cell derived cardiomyocytes (Parikh et al. 2017; Jackman et al. 2018). The Simonides lab has reported increased activity of the T3 inactivating enzyme, deiodinase type 3 (D3), in the failing remodeled ventricle after infarction that corresponds to reduced tissue T3 concentration and decreased T3-dependent transcription (Pol et al. 2011; Wassen et al. 2002; Simonides et al. 2008). Thus, we propose that reduced T3 activity in the failing myocardium affects T-tubule organization by acting either directly through genomic mechanisms in cardiomyocytes or indirectly through changes in myocardial hemodynamics and workload. Many excellent reviews have been published addressing the various mechanisms of action of THs in general and on the heart specifically (Gerdes and Ojamaa 2016; Jabbar et al. 2017; Janssen et al. 2014; Singh et al. 2018; Forini et al. 2019). Cardioprotective mechanisms of T3 action following MI include effects on sarcolemma ion currents, preservation of mitochondrial membrane potential with reduction of ROS generation, and activation of intracellular survival pathways leading to decreased cell death. In addition to numerous T3-responsive cardiac genes, recent studies have identified differentially expressed miRNAs in cardiac ischemia reperfusion injury and in pathologic cardiac hypertrophy that serve a protective role in response to T3 treatment (Janssen et al. 2014; Forini et al. 2018). Pertinent to the present study, Jph2 mRNA has been identified as a target of microRNAs, miR-24 and miR-34a, that have been shown to be increased in murine models of HF and pathologic hypertrophy, and in human failing myocardial tissue (Zhang et al. 2013; Xu et al. 2012; Hu et al. 2019). It remains to be determined whether T3 regulates expression of these miRNAs or activates Jph2 transcription directly.

As highly branched invaginations of the sarcolemma, the T-tubule system brings the L-type calcium channel (LTCC) in close proximity to ryanodine receptors (RyR, calcium release channels) in the sarcoplasmic reticulum, thereby regulating calcium flux with each depolarization (Bers 2002; Brette and Orchard 2003). Recently, the bridging integrator protein BIN1 has been shown to facilitate the microtubule-dependent delivery of LTCC to the T-tubule microdomains close to junctional SR containing RyR (Hong et al. 2010; Hong et al. 2012). To determine whether the observed changes in T-tubule periodicity would alter calcium kinetics and contractile function, we used the IonOptix imaging system that simultaneously measures calcium and contractility in single isolated cardiomyocytes. Supporting the hemodynamic changes observed in vivo, we recorded significant prolongation of time from initial electrical stimulus to peak myocyte shortening (i.e., contraction) in the failing cardiomyocytes, and T3 treatment significantly shortened this response time similar to that recorded in sham control myocytes. Furthermore, all measures of relaxation or relengthening of the failing myocytes, including maximum relaxation velocity and time from peak shortening to full relaxation, showed significant impairment, while T3 treatment normalized these parameters. Calcium transients recorded from these myocytes showed that time to peak Ca^2+^ tended to be shorter in T3 treated MI cardiomyocytes than untreated MI myocytes indicating faster kinetics. Differences were most evident in the declining phase of the Ca^2+^ transient including rate of Ca^2+^ fall and area under curve of the Ca^2+^ fall phase when cytosolic Ca^2+^ removal occurs primarily by the sarcoplasmic reticulum calcium-ATPase (SERCA2). The gene encoding SERCA2 has been documented as T3 responsive, and thus points to a potential genomic mechanism of T3 action in this setting (Gerdes and Ojamaa 2016). The RT-PCR analysis showed reduced SERCA2 mRNA expression in the MI hearts, with significant increases in SERCA2 expression with T3 treatment, thus supporting this mechanism of action. Rates of myocyte contraction also depend on the relative proportion of the contractile proteins, α- and β- myosin heavy chains, which exhibit either fast or slow contractile properties respectively. In animal models of heart failure, α-MHC expression has been shown to be decreased while the slower contractile isoform β-MHC is increased (Gerdes and Ojamaa 2016). Our data recapitulate these findings, and further show that T3 increased αMHC while βMHC expression was reduced, indicating that these genes are T3-responsive as has been well documented by many investigative groups (van Rooij et al. 2007; van Rooij et al. 2009).

To further interrogate the potential molecular events responsible for the effects of T3 in remodeling the T-tubule structures and the subsequent responses of myocyte function, we measured several TT and SR proteins. Expression of BIN1, Jph1, LTCC and RyR2 mRNAs were significantly decreased in the MI myocytes and normalized towards control levels with T3 treatment. BIN1 protein mirrored its mRNA expression pattern with a decrease in MI and significant increase with T3 treatment. Jph2 is a structural protein that serves to bridge the junctional SR and T-tubule structures, thereby maintaining association of the LTCC-RyR dyad and optimizing E-C coupling. In a mouse model of heart failure, the 97 kDa full length Jph2 protein was shown to be cleaved by calpain, a calcium-dependent protease, thus liberating a 75 kDa amino-terminal (NT)-fragment (Guo et al. 2015; Wu et al. 2014). Recently, Guo et al. (Guo et al. 2018) reported that the 75 kDa NT-fragment is imported into the nucleus where it associates with chromatin and regulates transcription of many genes resulting in attenuation of adverse remodeling in heart failure. Thus, although calpain-mediated cleavage of Jph2 in response to myocyte stress likely contributes to loss of TT-jSR structural integrity, the released smaller NT-fragment serves to protect the heart by reprogramming the transcriptome and thereby attenuating progression to failure. Our data show that the 97 kDa Jph2 protein in the microsomal fraction was largely unchanged with any treatment, whereas the 75 kDa fragment was higher in MI cardiomyocytes and further increased in T3-treated MI myocytes. These results appear to align with the recent findings of a cardioprotective role of the calpain-cleaved Jph2 NT-fragment. Hong et al. have shown that BIN1 not only folds the T-tubule membrane, but it also serves to deliver Ca_v_1.2 to the T-tubules by assisting its transport along microtubules (Hong et al. 2014; Hong et al. 2010). Immunoblot data showed that the RyR2 protein was increased in the micosomal fraction of MI myocytes with and without T3 treatment. The calcium transient data suggest improved coupling of RyRs with LTCC to form functional dyads in T3 treated myocytes, even though the total protein content was largely unchanged. Recent advancements in microscopic nanoimaging using STORM technology that allows visualization of single molecules or clusters of RyR and LTCC proteins at the junctional SR-TT microdomains will advance our understanding of the effects of T3 on the formation of the RyR-LTCC dyads in failing cardiomyocytes (Jayasinghe et al. 2018; Shen et al. 2019).

## Conclusions

We have shown that low dose T3 treatment maintained a functioning T-tubule network in a rat model of heart failure after myocardial infarction, resulting in significant improvements in cardiac function. Several questions remain including whether T3 treatment initiated after heart failure has been established can be effective in reversing TT dysfunction, and secondly, whether T3 treatment if initiated immediately after MI can be discontinued after ventricular remodeling is complete. T3-altered transcriptome or intracellular signaling pathways may be involved in maintaining T-tubule organization and processes (Hones et al. 2017). The present study results are further supported by recent published studies indicating a requirement of thyroid hormones in the early development and maturation of the cardiomyocyte T-tubule system (Parikh et al. 2017; Jackman et al. 2018). Therefore, the potential therapeutic utility of TH treatment in the setting of heart failure when subclinical hypothyroidism or low T3 syndrome is documented deserves full consideration.

## Data Availability

The datasets used and/or analyzed during the current study are available from the corresponding author on reasonable request.

## Abbreviations

BDM: 2,3-butanedione monoxime
BIN1: Bridging integrator-1 or amphiphysin 2
Ca_v_1.2: Calcium channel voltage-gated, pore-forming α-subunit
CVD: Cardiovascular diseases
DAPI: 4′,6-Diamidino-2-phenylindole
HF: Heart failure
Jph2: Junctophilin 2
LTCC: L-type calcium channels
LV: Left ventricle
MHC: Myosin heavy chain
MI: Myocardial infarction
PLN: Phospholamban
RyR2: Ryanodine receptor 2
SERCA: Sarcoplasmic reticulum calcium-ATPase
SR: Sarco-(endo) plasmic reticulum
T3: Triiodo-L-thyronine
T4: Thyroxine
TSH: Thyroid stimulating hormone
TT or T-tubule: Transverse-tubules
WGA: Wheat germ agglutinin
